# A Cohort Study of the Relationship between Active Collaboration and Operational Efficiency in Japanese Public Health Institutions

**DOI:** 10.31662/jmaj.2021-0195

**Published:** 2022-08-01

**Authors:** Motoi Miura, Tetsuya Tanimoto, Satoshi Miyata, Masayasu Murakami, Yoshinori Nakata

**Affiliations:** 1 Yamagata University Graduate School of Medical Sciences, Department of Health Policy Sciences, Yamagata, Japan; 2 Navitas Clinic Kawasaki, Kawasaki, Japan; 3 Teikyo University Graduate School of Public Health, Tokyo, Japan

**Keywords:** Japan, public hospital, efficiency, data envelopment analysis

## Abstract

**Introduction:**

In recent years, public hospitals have seen an increasing need for management reform in light of increasing social security costs due to the aging population. This study investigated the relationship between collaboration with neighboring medical institutions and management efficiency in public hospitals.

**Methods:**

Data envelopment analysis was used to calculate the dependent variable. We used the referral rate for each public hospital as an independent variable to indicate active collaboration. Univariate and multivariate analyses examined the association between the two variables above. The adjustment variables in the multivariate analysis incorporated those variables that were considered significant in the univariate analysis when the significance level was 10% on a two-sided basis. The Tobit regression model was used in both univariate and multivariate analyses.

**Results:**

Ultimately, the analysis included 402 public hospitals. Approximately 8% fell into the high-collaboration group. Even after adjusting for significant variables from the univariate analysis, the inefficiency value was significantly lower in the high-collaboration group than in the low-collaboration group; namely, the efficiency value in the high-collaboration group was significantly higher than in the low-collaboration group. Moreover, hospitals with a higher ratio of subsidies to revenue had significantly lower values for management efficiency.

**Conclusions:**

The analysis of the relationship between efficiency value and the percentage of referred patients in Japan indicated that higher percentages of referred patients, that is, higher degrees of cooperation, were significantly associated with higher efficiency scores.

## Introduction

Japan is known worldwide for providing high-quality medical services ^(1), (2)^. Japan’s medical system has universal health insurance and free access to care ^(3), (4), (5)^. As with other countries’ healthcare systems, public hospitals in Japan play an important role ^(6), (7)^. They provide standardized medical services even in unprofitable rural areas and unprofitable medical services, such as emergency medicine and pediatric care ^(8)^. However, in recent years, public hospitals have seen an increasing need for management reform due to increasing social security costs associated with an aging population ^(9), (10), (11), (12)^. The Ministry of Internal Affairs and Communications (MIC) issued guidelines for reforming public hospitals in 2007 and 2015, requiring each hospital to formulate a plan for reform and operational efficiency ^(13), (14)^.

According to a recent study, the factors affecting the management efficiency of public hospitals include the ratio of subsidies to revenue, nurse-to-patient ratio, hospital location, number of doctors and other medical staff, and medical revenue^(15), (16), (17), (18)^. In the new guidelines for public hospital (PH) reform, reviewing salary structure and reexamining departments were considered measures for improving business conditions, but these measures may be limited. Zhang et al. found no significant difference in management efficiency between PHs that could determine their salary structure and those that could not ^(19)^. Additionally, Chen et al. suggest that the provision of unprofitable care does not necessarily correlate with financial status ^(20)^.

However, in *Wealth of Nations*, Adam Smith concluded that regional cooperation promotes the division of labor and specialization of each entity and increases efficiency ^(21)^. To the best of our knowledge, no investigation has been conducted into whether active collaboration with neighboring medical institutions has affected the management efficiency of PHs. This study has examined the relationship between collaboration with neighboring medical institutions and management efficiency in PHs. Moreover, as an indicator of the degree of collaboration (DOC) with other facilities, we adopted the referral rate, which several organizations use to indicate the degree of active collaboration ^(22), (23)^.

### Hypothesis

We hypothesize that PHs that actively collaborate with neighboring hospitals (NH) are more efficient in their operations than those that do not.

## Materials and Methods

### Research subjects and eligibility criteria

The inclusion criteria for facilities were: hospitals listed in the FY2019 Local Public Enterprise Yearbook (LPEY) ^(24)^ published by the MIC; hospitals that adopt the diagnosis procedure combination (DPC) system charging a fixed amount of medical expenses per day, which accounts for all procedures, medication, etc., at a rate that matches the name of the hospitalized patient’s disease; hospitals preparing for the adoption of the DPC system; hospitals that use a fee-for-service system to submit DPC data to the Ministry of Health, Labor and Welfare (MHLW) ^(25)^; and hospitals listed in the survey of medical facilities published by the MHLW for FY2019 ^(26)^. The exclusion criteria, in line with previous studies, were: hospitals specializing in tuberculosis, psychiatry, and other specialties (cancer treatment, rehabilitation, rheumatism, and cardiovascular diseases) were excluded ^(19)^; hospitals with a ratio of general hospital beds to total hospital beds of less than 80%; and hospitals where the average length of stay was longer than 70 days or shorter than 6 days.

### Dependent variable

We adopted the technical inefficiency value as the dependent variable, consistent with previous studies. The technical inefficiency value was the inverse of the technical efficiency value minus one, calculated using data envelopment analysis (DEA)―used for calculating efficiency values among decision-making units (DMUs); DEA has been used in many studies in medicine ^(27), (28), (29), (30), (31), (32), (33)^. The DEA method is explained in detail in the supplementary file. Further, an efficient PH used the least amount of human, financial, and material resources to care for a certain number of inpatients and outpatients. From this definition and because previous studies have shown that PHs may have increasing returns to scale, we adopted the input-oriented Banker-Charnes-Cooper model to calculate the efficiency value ^(34)^.

Owing to our focus on the management efficiency of PHs, we designated DMUs as the research target hospitals. The outputs and inputs of the DMUs were based on the previous study, which explored the factors that determine the management efficiency of PHs in Japan ^(19)^. First, the outputs were defined as the number of per day outpatients and inpatients. The inputs were the number of doctors, nurses, and other medical professionals and the number of hospital beds and materials costs. The data came from the FY2019 LPEY and the FY2019 Hospital Bed Function Report published by the MHLW ^(24), (26)^. We used these inputs and outputs to calculate the efficiency of each PH. We used DEA Solver Pro 12.1 (Saitech, Inc., Tokyo, Japan) to perform the calculations. The target hospitals were assigned a technical efficiency value between 0 and 1, and PH with the highest management efficiency was assigned an inefficiency score of 0.

### Independent variable

We took the referral rate (RR) of each PH as an independent variable indicating active collaboration. The RR was the number of first-time patients referred from other hospitals per year, divided by the number of all first-time patients minus the number of patients transported by ambulance on holidays and at night, together with the number of first-time emergency patients ^(35)^. This data were drawn from MHLW’s report “Survey on the evaluation of the impact of the introduction of DPC in fiscal 2019: Aggregate results” ^(25)^.

Facilities with an RR of 80% or higher were the high-collaboration group and assigned a dummy variable value of 1. The other facilities were designated as the low-collaboration group and assigned a value of 0. To create categorical variables, we used the requirement of an 80% or higher RR, which is a requirement for certification as a community medical support hospital in Japan. A community medical support hospital is a medical institution that supports primary care doctors to promote cooperation among medical institutions in the community and should show a significant degree of strong cooperation with NHs ^(36)^.

### Control variables

Previous studies selected four variables that affected management efficiency in PHs as control variables.

#### 1. The area where the PH is located

This variable was the region where the PH was located. Previous research has found that in some regions of Japan, PHs are the main source of healthcare services, and there, the impact of the reform plan is greater than it would be in other regions ^(19)^. The region of the hospital’s location was tracked to derive the results that adjust for these effects. Then, following previous studies, we divided the regions of Japan into three categories ^(19)^.

The regions were classified into three groups. Japan is usually divided into eight main regions, i.e., Hokkaido, Tohoku, Kanto, Chubu, Kinki, Chugoku, Shikoku, and Kyushu. Hospitals located in Hokkaido and Tohoku were defined as the East Group and assigned the dummy variable value 1 for location. Hospitals located in the Kanto, Chubu, and Kinki regions were defined as the Central Group and assigned 2 for the dummy variable. Finally, Chugoku, Shikoku, and Kyushu hospitals were defined as the West Group and assigned 3. The data used in this study were from the FY2019 LPEY ^(24)^.

#### 2. Percentage of subsidies from the government

Subsidies percentage was calculated by dividing the number of government subsidies by the current account balance. Previous studies have found that high levels of financial subsidy from the government lead hospital managers to invest in useless equipment regardless of demand, producing inefficiency ^(37)^. This factor was included in the model to adjust for its effect on efficiency. For this variable, we used data on the ratio of the current account balance to transfers to other accounts in the FY2019 LPEY ^(24)^.

#### 3. Average length of stay

This variable represents the average time between admission and discharge. Previous studies have shown that longer average lengths of stay result in lower bed occupancy and less efficiency ^(38)^. This variable was used as an adjustment variable to exclude the effect of this factor. The variable was obtained from the FY2019 LPEY ^(24)^.

#### 4. Population density of the municipality wherein PH is located

The population density of the municipality variable is the population per 1,000 km^2^ of the municipality where the hospital is located. Previous studies showed that high population density and the associated need for a variety of medical care might increase the demand for medical services and result in a greater variety of medical services in PHs, thus increasing the operational efficiency of PHs ^(39)^. This variable was adopted to adjust for the effects of population density on efficiency. Data from MIC’s 2015 census, the most recent census before 2019, were used to compute this variable ^(40)^.

#### Statistical analysis

First, summary statistics were calculated to investigate the dependent, independent, and adjustment variables. The Mann-Whitney U test was used to assess the continuous variables, and the chi-square test was used for categorical variables to investigate whether there was a significant difference between the values of the high-collaboration group and the low-collaboration group for the collected variables.

Next, for each combination, a simple linear regression model was used to conduct univariate analysis. The dependent variable was the technical inefficiency value and the independent variables were dummy variables based on the RR or variables obtained as adjustment variables.

Finally, a multivariable linear regression model was used for a multivariate analysis using the technical inefficiency value as the dependent variable, RR as the independent variable, and a variable found to be significant when the significance level of the univariate analysis was P < 0.1 as adjustment variable. A logarithmic transformation was performed if the Shapiro-Wilk test did not normally distribute the adjustment variables. Furthermore, in both the multivariate and univariate analyses, the dependent variable drew on censored data; hence, the Tobit model was used in line with previous studies ^(41)^. A two-tailed significance level of 5% was used for analysis, conducted using STATA 14.0 (State Corporation, Lake Way, Texas, USA).

### Ethical issues/Statement

Because all data in this study were publicly available, the application for ethical approval was waived.

## Results

**Figure 1.**
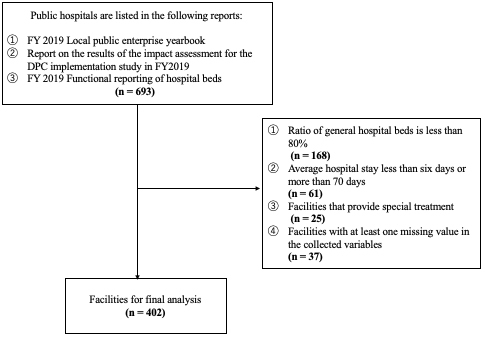
Selection process of subjects of the analysis.

There were 693 PHs listed in the FY2019 LPYE that submitted DPC data and responded to the FY2019 Hospital Bed Function Report. After applying the exclusion criteria, 254 facilities were excluded. Moreover, assuming that the missing data occurred randomly, 37 facilities with missing data in one or more variables were excluded from the analysis. Ultimately, 402 PHs were included in the analysis, as shown in Figure 1.

The characteristics of the hospitals subjected to analysis are shown in Table 1. Using the Mann-Whitney U test for continuous variables and the chi-squared test for categorical variables, comparisons between high- and low-collaboration groups were performed. Approximately 8% of hospitals were included in the high-collaboration group. There were significant differences between the high- and low-collaboration groups in terms of the ratio of subsidies to revenue, the average length of stay, and the population density of the facility site. As shown in Table 2, DOC, percentage of subsidies, location, and population density had significant relationships with inefficiency values for significance level at P < 0.1. Furthermore, as indicated in Table 2, even after adjusting for the variables that were significant in the univariate analysis as adjustment variables, the inefficiency value was significantly lower for the high-collaboration group than for the low-collaboration group (coefficient: −0.12, 95% confidence interval: −0.21, −0.03, P = 0.01), that is, the efficiency value of the high-collaboration group was about 0.14 points higher than that of the low-collaboration group.

**Table 1.** Analysis Subject Characteristics.

Variable	All (n = 402)	High-collaboration group (n = 32)	Low-collaboration group (n = 370)	Test statistics	P-value
Inefficiency score (mean, SD)	0.83 (0.12)	0.83 (0.12)	0.91 (0.06)	3.97	0.0001
Areas with public hospitals (n)				0.92	0.63
1. Hokkaido, Tohoku	95 (24)	7 (22)	88 (24)		
2. Kanto, Chubu, Kinki	208 (52)	19 (59)	189 (51)
3. Chugoku, Shikoku, Kyusyu, Okinawa	99 (24)	6 (19)	93 (25)
Percentage of subsidies from government (%) (median, Q1, Q3)	11.0 (7.7, 17.2)	8.1 (6.6, 11.7)	11.2 (7.8, 18.0)	2.85	0.0044
Average length of stay (days) (mean, SD)	15.4 (5.4)	13.1 (7.4)	15.6 (5.2)	5.29	<0.00001
Population density (person/square kilometer) (median, Q1, Q3)	262.9 (93.0, 965.4)	1093.4 (711,3, 4683,9)	220 (83.6, 740.0)	−5.34	<0.00001

Comparisons between high- and low-collaboration groups, conducted using the Mann-Whitney U test for continuous variables and the chi-squared test for categorical variables.

**Table 2.** Results of Univariate and Multivariate Analysis.

	Results of univariate analysis^1^						Results of multivariate analysis^2^					
Variable	Coefficient	Standard error	T-value	95% confidence interval		P-value	Coefficient	Standard error	T-value	95% confidence interval		P-value
				Lower	Upper					Lower	Upper	
Degree of collaboration											
0. Low-collaboration group	Reference						Reference					
1. High-collaboration group	−0.14	0.05	−3.00	−0.23	−0.05	0.03 × 10^−1^	−0.12	0.05	−2.55	−0.21	−0.03	0.01
Log (percentage of subsidies from government)	0.22	0.04	5.28	0.14	0.30	<0.01 × 10^−2^	0.20	0.04	4.42	0.11	0.28	<0.01 × 10^−2^
Log (average length of stay)	0.06 × 10^−3^	0.10	0.60	−0.14	0.26	0.55						
The public hospital area												
1. Hokkaido, Tohoku vs. 2. Kanto, Chubu, Kinki	−0.09	0.03	−3.01	−0.15	−0.03	0.03 × 10^−1^	−0.07	0.03	-1.98	-0.13	-0.03 × 10^−1^	0.05
1. Hokkaido, Tohoku											
vs. 3. Chugoku, Shikoku, Kyusyu, Okinawa	0.01	0.04	0.37	−0.06	0.08	0.71	0.03	0.03	1.00	−0.03	0.10	0.32
2. Kanto, Chubu, Kinki										
vs. 3. Chugoku, Shikoku, Kyusyu, Okinawa	0.10	0.03	3.47	0.05	0.16	0.001	0.10	0.03	3.29	0.04	0.16	0.01 × 10^−1^
Log (population density)	−0.04	0.02	−2.09	−0.07	−0.02 × 10^−1^	0.04	0.01	0.02	0.48	−0.03	0.05	0.16

1: A simple linear regression model was used to conduct univariate analysis for each combination, with the dependent variable being technical inefficiency value and the independent variables being dummy variables based on the referral rate (RR) or variables collected as adjustment variables. 2: A multivariable linear regression model was used to conduct multivariate analysis with technical inefficiency value as the dependent variable, RR as the independent variable, and a variable found to be significant when the significance level of the aforementioned univariate analysis was set at P < 0.1 as adjustment variable.

## Discussion

In this retrospective cohort study in Japan, using DEA, we observed a significant relationship between the DOC with NHs and the operational efficiency of PHs, even after adjusting for the factors suggested by previous studies as possible influences on the operational efficiency of PHs. In other words, our results suggest that increased percentages of referred patients and increased degrees of collaboration with NHs may improve management efficiency in PHs.

To the best of our knowledge, this is the first study to investigate whether collaboration with NHs affects management efficiency in PHs. Although the results may not be universally applicable due to differences in healthcare delivery systems and institutions among countries and regions, many studies have investigated factors affecting the management efficiency of PHs in Japan and other countries. In Japan, one study concluded that the hospital’s region affects the management efficiency of PHs and the operational efficiency of their dispensing departments ^(19)^. Similar studies have been conducted in other countries; for example, a study that explored the factors determining the efficiency of PHs in Uganda found that bed utilization and the number of beds affected efficiency scores ^(42)^. Furthermore, a study that examined the impact of the transfer of PHs in Germany to private operators on management efficiency found that the transfer to the private sector resulted in significantly more efficient management ^(43)^. However, as far as we know, in Japan and elsewhere, few studies have investigated the relationship between the DOC with NHs and management efficiency in PHs.

The results herein are in line with the findings of previous work in that the ratio of subsidies to revenue has been found to affect management efficiency in PHs ^(37)^. In this study, hospitals with a higher ratio of subsidies to revenue had significantly lower values for management efficiency. Moreover, as with previous studies, the regional location of the hospital had a significant effect on operating efficiency ^(19)^. However, unlike these studies, we conclude that the average length of stay, a factor found to most affect efficiency in our study, may not have had a significant impact ^(38)^. We speculate this is mostly because there was little difference in the average length of stay among the facilities in our study. The target was hospitals had adopted or were planning to adopt the DPC system, designed to limit profitability as the number of days spent in the hospital increases.

Further, population density, which showed a significant relationship to efficiency in a previous study conducted in a PH in Saudi Arabia, was not found to be significantly associated with efficiency ^(39)^. We believe that this may be due to the free access provided by the Japanese healthcare system. Previous studies have concluded that high population densities and related needs for various medical care trigger the accelerated use of medical services. Existing hospitals in the area have increased their production of medical services to respond to these needs, resulting in higher management efficiency. However, even if a wide range of demands for medical services occurs in a densely populated area, if this principle of free access exists, the place to receive medical services may fall outside the area where the demand occurs. If this happens, hospitals in the region where the demand occurs need not increase their production of medical services, and productivity will not increase. Therefore, the relationship between location in high population density areas and efficiency, shown in previous studies, did not hold because the demand for medical services generated in high population density areas was then met in other areas. The supply of medical services in high population density areas did not change due to the existence of the free access system.

Our study has important implications for future policy considerations regarding PHs in Japan. Around 92% of Japanese PHs have an RR lower than 80%. Increasing this rate, even if only slightly, and increasing the level of cooperation between NHs may increase the management efficiency of PHs. To improve management efficiency, making large-scale changes, such as investing in new medical equipment or changing operating forms of operation, is inessential; instead, management can be improved by strengthening cooperation with similar facilities in the neighboring areas. Furthermore, according to the results of the multivariate analysis, a decrease in the ratio of subsidies to revenues may also create improved management efficiency in PHs.

It is important to investigate whether other indicators of the DOC with other medical institutions affect management efficiency in PHs. Some potential indicators would include the reverse RR, or the percentage of first-time patients referred to other appropriate medical institutions due to their disease status; the number of patients using the community collaborative clinical path developed to create medical treatment plans for multiple facilities within the same region, from acute care hospitals to recovery hospitals, and to share them among all medical institutions providing treatment; the number of collaborating doctors; and the amount of expensive medical equipment shared with other institutions. An important tool for hospital managers to check the management efficiency of their hospitals could be created if the relationship between these variables and management efficiency could be clarified.

We expected higher efficiency values through functional differentiation and specialization in medical services, exploiting comparative advantage through collaboration with NHs. A field of comparative advantage is one where productivity is relatively high within an entity, and specializing in this field can increase productivity ^(44)^. Future research should verify these detailed mechanisms after an empirical analysis of how resources, such as material costs and the number of staff, can be input to provide medical services and how the services have changed due to functional differentiation.

There are several limitations of this study. First, the definition of efficiency value may change the results. In this study, the inputs for calculating efficiency values were defined as the number of doctors, nurses, and other medical professionals, total hospital beds, and material costs. The outputs were defined as the daily number of inpatients. However, an exploratory study of the factors that determine the management efficiency of Palestinian PHs set the outputs to the total number of inpatient days per year, the number of outpatients per year, and the number of emergency patients received, while the inputs were the number of beds, doctors, and other staff ^(45)^. In another study, which investigated the management efficiency of PHs by province―defining each DMU as a province―the inputs were the total number of medical personnel, the total number of hospital beds, and number of PHs in the province. The outputs were the number of outpatients and inpatients and the occupancy rate of the beds ^(46)^.

Second, we ignored patients’ images of the hospitals. While calculating efficiency values and in univariate and multivariate analyses, the patient profile of each hospital was considered to be the same. In general, hospitals at smaller scales tend to treat relatively easier cases; thus, fewer resources are needed to treat one patient. Therefore, efficiency values may have been overestimated at smaller relative to larger hospitals.

Third, we may have missed some unexpected confounders. We determined the confounding factors for multivariate analysis based on an extensive review of the existing literature, but it is possible that we did not cover all of them. If such factors are present and considered in the analysis, the results of this study would change.

Fourth, for small-sized PHs, RR may not accurately reflect the DOC with surrounding hospitals. This is because some small-sized hospitals provide general practitioner medical care, and in such cases if they actively collaborate with NHs, the aforementioned reverse RR will increase ^(47)^. In other words, active collaboration and an increase in RR may not be equivalent for small- and medium-sized hospitals. We may have underestimated the DOC in small hospitals.

The fifth limitation is that RR may be an intermediate factor. In other words, environmental factors, such as location, may have an impact on RR. Even if RR is an intermediate factor, we believe that this study is very meaningful in clarifying the mechanism through which environmental factors affect the management efficiency of PHs.

As a result of analyzing the relationship between the efficiency value calculated using DEA and the percentage of referred patients in Japan, which indicates the DOC with NHs in PHs, we show that the higher the percentage of referred patients. The higher the DOC is, the higher the efficiency score, to a significant degree. This result provides important evidence for planning policies to improve the business condition of PHs, which has become an important issue in Japanese society in recent years.

## Article Information

### Conflicts of Interest

Dr. Tanimoto reports personal fees from Medical Network Systems, MNES Inc., and Bionics Co., Ltd., outside the submitted work.

### Author Contributions

Motoi Miura: conception of the study design, analysis of the data, interpretation of the analysis results, draft of the article, and final approval of the article; Tetsuya Tanimoto: critical revision of the article about the interpretation of analysis results and final approval of the article; Satoshi Miyata: analysis and interpretation of the data and final approval of the article; Masayasu Murakami: critical revision of the article about the interpretation of analysis results and final approval of the article; and Yoshinori Nakata: conception of the study design, critical revision of the article about the interpretation of analysis results and final approval of the article.

### Approval by Institutional Review Board (IRB)

Because all data in the present study comprised publicly available data, the ethical approval application was waived.

## Supplement

Supplementary Material 1

Supplementary figure 1

Supplementary figure 2
