# Supplementary material for: A Cohort Study of the Relationship between Active Collaboration and Operational Efficiency in Japanese Public Health Institutions
Source: JMA J. 2022 Aug 1;5(4):438–45. doi: 10.31662/jmaj.2021-0195 (PMC9646289; doi:10.31662/jmaj.2021-0195)
Supplement: Supplementary file 1 — Supplementary Material 1 [file 2433-3298-5-4-0438-s001.pdf]

In the flowing, we briefly discuss the structure of the efficiency calculation method in DEA. There are two types of DEA: input-oriented models and output-oriented ones. First, we will report how efficiency values are calculated in an input-oriented model. This model assumes that the DMU operates efficiently by changing the level of input, given a certain level of output. In an input-oriented model, the efficiency value was calculated using an isoquant that represents how a certain amount of output was achieved with a minimum combination of input levels. As an example, Supplementary figure 1 presents the isoquant for the case where one type of output was produced using two types of inputs.

The highlighted region is the set of points giving the feasible input combinations. In this case, points on the line QQ’ give the most efficient combination of inputs. The efficiency value of inefficient point A can be expressed as OA’/OA, where A’ is the intersection of the line drawn from the origin O to A and the line segment QQ’. Similarly, the efficiency value of the efficient point B can be expressed as OB’(=OB)/OB, or 1.

The next section reports the efficiency values for the output-oriented model. This model assumes that the DMU operates efficiently by changing the output level, given a certain level of input. Supplementary figure 2 presents the production possibility frontier for cases where two types of outputs are produced using one type of input as an example. The highlighted region provides the set of points that give the possible output combinations. The points on the line RR’ are the most efficient combinations of outputs. The efficiency value of the inefficient point C can be expressed as OC’/OC, where C’ is the intersection of the line drawn from the origin O to C along line segment RR’. The efficiency value of the efficient point D can also be expressed as OD’(=OD)/OD, or 1.

These two models can be classified into two types, depending on their assumptions about scale. Specifically, the Charnes–Cooper–Rhodes model assumes constant returns to scale, and the Banker–Charnes–Cooper model assumes diminishing or increasing returns to scale.

Figure legends

Supplementary figure 1. The isoquant for the case where one type of output was produced using two types of inputs.

Supplementary figure 2. The production possibility frontier for cases where two types of outputs were produced using one type of input.
