# Supplementary figures and images for: A Cohort Study of the Relationship between Active Collaboration and Operational Efficiency in Japanese Public Health Institutions
Source: JMA J. 2022 Aug 1;5(4):438–45. doi: 10.31662/jmaj.2021-0195 (PMC9646289; doi:10.31662/jmaj.2021-0195)

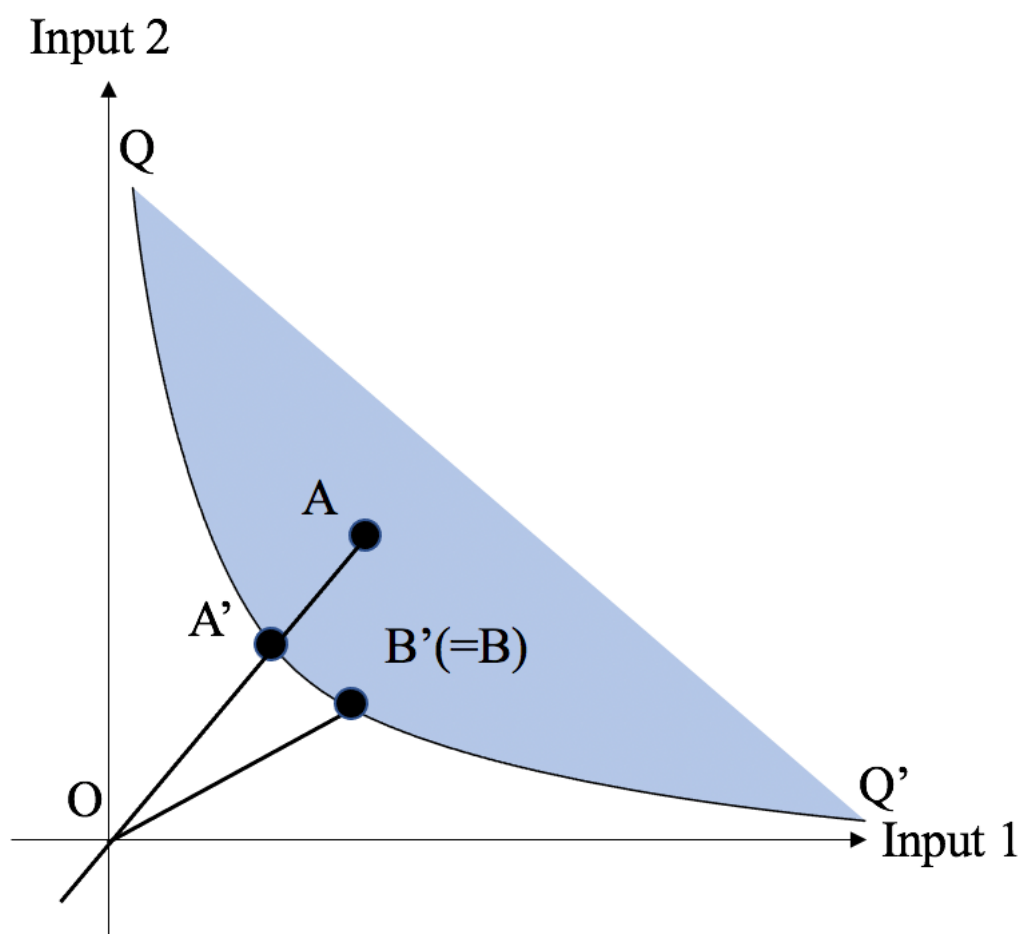

Supplement: Supplementary file 2 — Supplementary figure 1 [file 2433-3298-5-4-0438-s002.pdf]

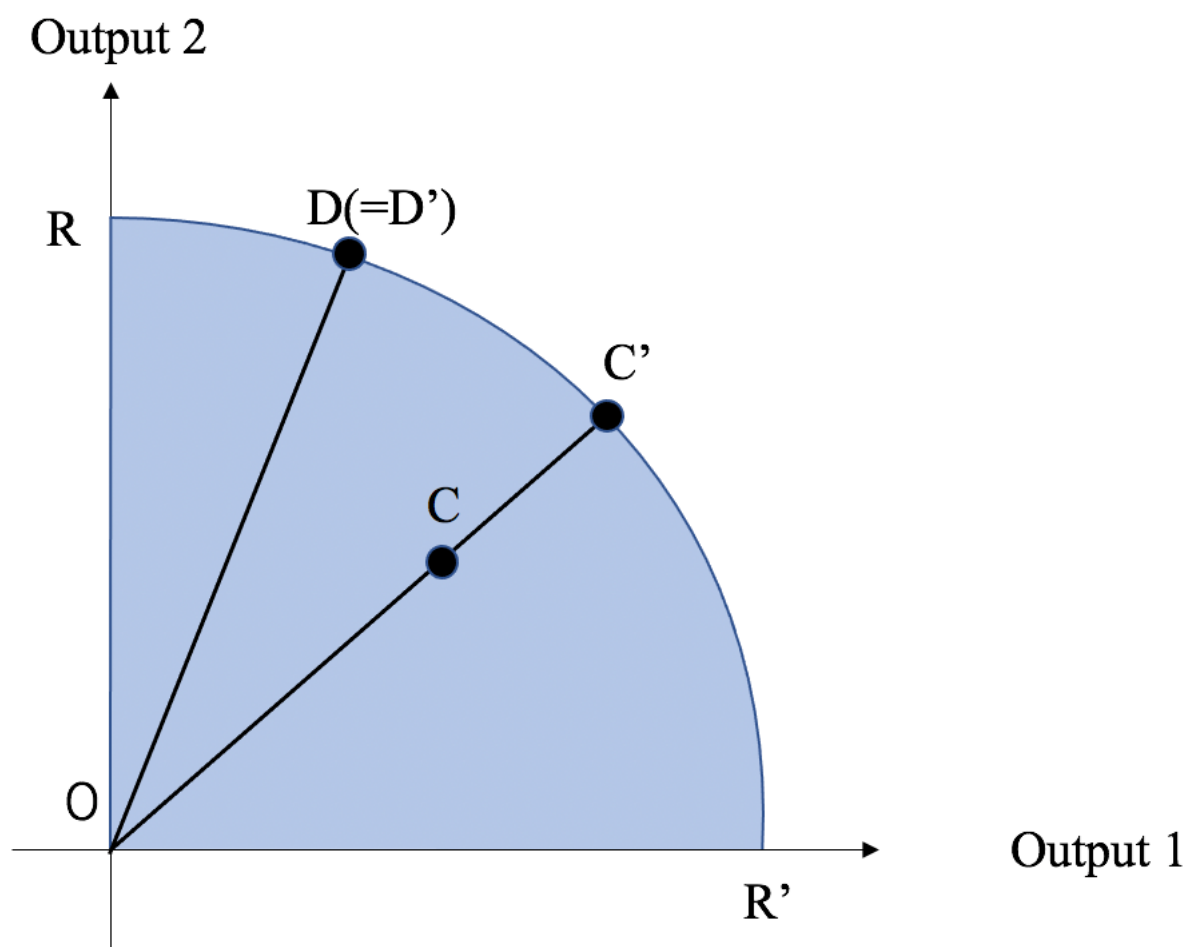

Supplement: Supplementary file 3 — Supplementary figure 2 [file 2433-3298-5-4-0438-s003.pdf]
